# Usual Nutrient Intakes from the Diets of US Children by WIC Participation and Income: Findings from the Feeding Infants and Toddlers Study (FITS) 2016

**DOI:** 10.1093/jn/nxy059

**Published:** 2018-06-05

**Authors:** Shinyoung Jun, Diane J Catellier, Alison L Eldridge, Johanna T Dwyer, Heather A Eicher-Miller, Regan L Bailey

**Affiliations:** 1 Department of Nutrition Science, Purdue University, West Lafayette, IN; 2 RTI International, Research Triangle Park, NC; 3 Nestlé Research Center, Lausanne, Switzerland; 4 Tufts University School of Medicine, Boston, MA

**Keywords:** B-24, Feeding Infants and Toddlers Study, FITS, WIC, usual nutrient intake, pediatric nutrition

## Abstract

**Background:**

A recent report of the National Academies of Sciences, Engineering, and Medicine (NASEM) outlined priority nutrients for infants and children participating in the Special Supplemental Nutrition Program for Women, Infants, and Children (WIC).

**Objective:**

The objective of this study was to assess usual nutrient intakes from foods and beverages (not supplements) among US children aged <4 y by WIC participation status.

**Methods:**

A national random sample of children aged <4 y (*n* = 3,235) from the Feeding Infants and Toddlers Study (FITS) 2016 was categorized by WIC participation status (participants, lower-income nonparticipants, or higher-income nonparticipants) and age (younger infants aged 0–5.9 mo, older infants aged 6–11.9 mo, toddlers aged 12–23.9 mo, or preschoolers aged 24–47.9 mo). All participants contributed one 24-h dietary recall, with a second recall from a representative subsample (*n* = 799). Usual intakes and compliance with federal dietary recommendations were estimated by using the National Cancer Institute method. Differences between WIC participants and either lower-income nonparticipants or higher-income nonparticipants were tested using *t* tests.

**Results:**

The diets of infants (aged <12 mo) were nutritionally adequate in general. Older infants participating in WIC had higher compliance with iron and vitamin D guidelines than either group of nonparticipants and greater compliance with calcium, zinc, and potassium guidelines than higher-income nonparticipants. WIC toddlers had a higher risk of inadequate calcium and excessive sodium intakes than higher-income nonparticipants. Eight percent of WIC toddlers exceeded added sugar guidelines compared with either nonparticipant group (∼2%). WIC toddlers and preschoolers had a lower risk of inadequate vitamin D intake than lower-income nonparticipants, but inadequacy was >75% across all subgroups. WIC preschoolers had higher compliance with saturated fat guidelines but lower compliance with sodium and added sugar guidelines than higher-income nonparticipants.

**Conclusions:**

WIC participants had better intakes of iron (ages 6–23.9 mo), zinc and potassium (ages 6–11.9 mo), saturated fat (ages 24–47.9 mo), and vitamin D (all ages). Regardless of WIC participation status, most infants and children met the calcium and zinc guidelines, but large proportions had intakes not meeting the recommendations for iron (ages 6–11.9 mo), vitamin D, potassium, fiber, saturated fat, and sodium.

## Introduction

Approximately 1 in 5 US children lives in poverty (1). Lower socioeconomic status increases the likelihood of suboptimal nutritional intakes (2–4). In 1975, US Congress established the Special Supplemental Nutrition Program for Women, Infants, and Children (WIC) as a federal program “to safeguard the health of low-income women, infants and young children.” The reach of the WIC program is extensive, with ∼1.9 million infants and

4.2 million young children age <5 y participating in fiscal year 2015 (5). On average, approximately half of all infants in the United States and more than one-quarter of children <5 y old participate in WIC (5).

WIC provides free food packages, nutrition education, and health care referrals to pregnant and postpartum women, infants, and young children age <5 y who are in low-income households and at nutritional risk. WIC food packages are tailored to supplement dietary intakes, specific to age group or life stage (i.e., pregnancy). In 2009, significant changes to the WIC food package were made for the first time since its implementation. The package was revised to align more fully with the 2005 Dietary Guidelines for Americans and the infant feeding practice guidelines of the American Academy of Pediatrics, as well as to address concerns about the high prevalence of childhood obesity (6, 7). The revised food packages include more fruits, vegetables, whole grains, and lower-fat milk, and less juice than previous packages (8). Dietary supplements are not included in the WIC food package.

Little information is available on the nutrient intakes of infants and young children participating in WIC after the 2009 changes in the WIC food package. The nation's population-based survey, NHANES, does not report dietary intake estimates of breastfed infants, and the sample size of young children indicating participation in WIC is limited (8). Moreover, to our knowledge, there are no recent large, comprehensive studies that have investigated the diets of WIC infants and toddlers. The Feeding Infants and Toddlers Study (FITS) 2016 provides detailed dietary information and WIC participation status on a national sample of children from birth to age 4 y (9, 10). The objective of this study was to assess usual dietary intakes from foods and beverages among US children age <4 y by WIC participation status (current participants, lower-income nonparticipants, and higher-income nonparticipants) with the use of the FITS 2016 data, with a special focus on priority nutrients identified by the National Academies of Sciences, Engineering, and Medicine (NASEM) (8).

## Methods

### 

#### FITS survey methods

The FITS 2016 is a nationwide, cross-sectional study in parents or caregivers of children, from birth up to the age of 4 y, living in the 50 United States and Washington, DC. Data were collected from 4 sampling frames designed to cover the US population, and the resulting sample was weighted and calibrated to the US 2014 Census divisions, accounting for child age, WIC status, race/ethnicity, and educational attainment of the parent or caregiver. The FITS 2016 followed 2 previous FITS surveys conducted in 2002 and 2008 (11, 12). Full details of the study methodology for FITS 2016 are available elsewhere (10).

Demographic characteristics, including sex and race/ethnicity, were assessed with the use of questionnaire data; dietary supplement use was assessed by using dietary recall interview data. The 24-h dietary recalls were collected by telephone by trained interviewers from the University of Minnesota's Nutrition Coordinating Center with the use of the Nutrition Data System for Research (version 2015; University of Minnesota). A random subsample of 25% of the total sampled population underwent a second 24-h recall (*n* = 799), of whom 275 were children participating in the WIC program. For volume of breastmilk consumed via breastfeeding, not fed in a bottle, coding rules established for FITS 2008 were applied according to the age of the child and whether the child was exclusively or partially breastfed, as in previous FITS surveys (12–16). Briefly, exclusively breastfed younger infants aged 0–5.9 mo and older infants aged 6–11.9 mo were assigned breast-milk volumes of 780 and 600 mL/d, respectively. Partially breastfed younger and older infants were assigned breast-milk volumes by subtracting the amount of formula or other milks consumed from the breast-milk volume assigned for exclusive breastfeeding. Breastfed toddlers aged 12–17.9 mo and young children aged 18–47.9 mo were assigned breast-milk volumes of 89 mL/feeding occasion and 59 mL/feeding occasion, respectively. All study instruments were pilot tested before use and were available in English and Spanish. The final instruments were reviewed and approved by the institutional review boards of RTI International, the University of Minnesota Nutrition Coordinating Center, and the Docking Institute of Public Affairs, Fort Hays State University. Data were collected from June 2015 to May 2016.

Stratified random sampling with targeted oversampling in 0- to 17.9-mo-olds was used to achieve prespecified sample size targets for age and WIC participants (*n* = 1161) and nonparticipants (*n* = 2068). For analysis, the nonparticipant group was further divided into 2 subgroups: lower-income (and likely WIC eligible) nonparticipants (*n* = 641) and higher-income (and likely WIC ineligible) nonparticipants (*n* = 1427). Children aged <5 y are eligible for WIC if their family's income is <185% of the federal poverty guidelines (which depend on household size) and if they are at nutritional risk. However, because family income data were collected in ranges, the income eligibilities were estimated for those not participating in WIC on the basis of the reported income range, household size, and the WIC income eligibility cutoffs. Ages were categorized as young infants (0–5.9 mo), older infants (6–11.9 mo), toddlers (12–23.9 mo), and preschoolers (24–47.9 mo).

#### Statistical analysis

Means and usual intake distributions of energy, macronutrients, and selected micronutrients were computed with the use of the National Cancer Institute (NCI) method (17). The NCI method partitions out the within-person (day-to-day) component of variation in reported intakes when estimating the distributions of intakes, and therefore requires replicate measures on at least a representative subsample. With replicate measures from a representative subsample (∼25%), we were able to estimate usual intakes across all age groups. The covariates in the NCI method macros included the sequence and the day of the week on which the 24-h dietary recall was collected, dichotomized as weekend or weekday.

The nutrients of interest were selected on the basis of the priority nutrients identified by a 2017 NASEM report (8) and included iron, zinc, calcium, vitamin D, fiber, and potassium as nutrients to increase and sodium, saturated fat, and added sugar as nutrients to limit when federal dietary recommendations are available for specific age groups. We compared usual mean intakes and adherence to the appropriate DRI to assess the likelihood of nutrient inadequacy or excess following methods recommended by the Food and Nutrition Board of the Institutes of Medicine (18). For nutrients with an Estimated Average Requirement (EAR), the percentile of the usual intake distribution below the EAR was used to estimate the percentage of children in the population predicted to be “at risk for nutrient inadequacy.” For nutrients with an Adequate Intake (AI), the percentile exceeding the AI was used to estimate the percentage of children predicted to be “at low risk of inadequacy.” Sodium intakes in children aged ≥12 mo were compared with the Tolerable Upper Intake Level (UL), and the percentile of the intake distribution exceeding the UL was used to estimate the prevalence of excessive intake. For macronutrients, it is recommended that mean intakes should fall within the Acceptable Macronutrient Distribution Range, expressed as a percentage of total energy intake. For added sugars, the NASEM guidelines are to limit intake to <25% of total energy for children aged ≥12 mo. In addition, the 2015–2020 Dietary Guidelines for Americans recommend that saturated fat consumption should be <10% of total energy for those aged ≥24 mo (19). The percentages of children consuming added sugars and saturated fat at amounts above the recommendations were estimated. Only AIs are available for infants aged <6 mo and therefore they were not part of this analysis for DRI compliance.

All of the statistical analyses were performed on weighted data with the use of SAS software (version 9; SAS Institute, Inc.) and SAS-callable SUDAAN (version 9; RTI International). Differences in demographic characteristics and dietary supplement use were determined by using chi-square tests. Multiple *t* tests were used to compare mean intakes and compliance with DRIs between WIC participants and either lower-income or higher-income nonparticipants within age group. Significance was considered at *P* < 0.05 unless otherwise noted; a Bonferroni-corrected *P* value of 0.002 was used to compare mean intakes presented in **Supplemental Tables 1–4**.

## Results

### 

#### Demographic characteristics and dietary supplement use

**FIGURE 1:**
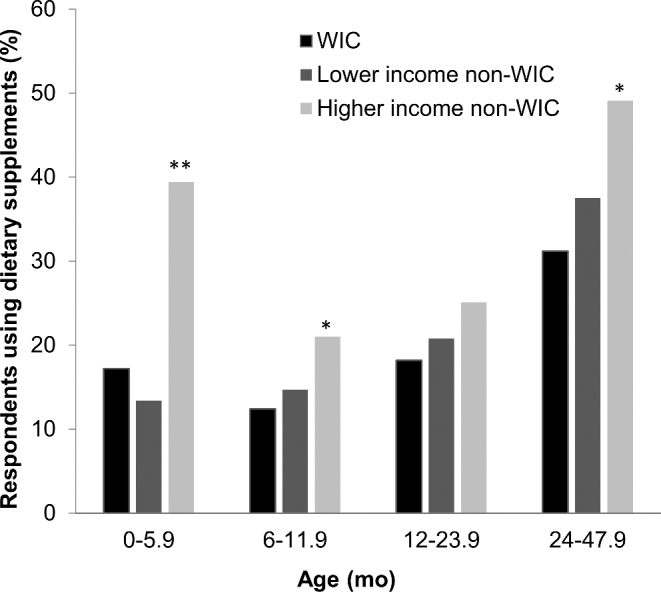
Prevalence of dietary supplement use by WIC participation and income: FITS 2016. Chi-square tests for differences across WIC categories within age group were conducted. *P < 0.05, **P < 0.001. FITS, Feeding Infants and Toddlers Study; WIC, Special Supplemental Nutrition Program for Women, Infants, and Children.

WIC participation was higher in 0- to 11.9-mo-olds (∼40%) than in 12- to 23.9-mo-olds (34%) and 24- to 47.9-mo-olds (27%). A detailed description of the FITS 2016 sample characteristics by WIC participation and income is published elsewhere (20). Briefly, approximately half of WIC participants were in households also receiving Supplementary Nutrition Assistance Program (SNAP) benefits, which is significantly higher than nonparticipants in lower-income households (27%). Across all ages, WIC participants were less likely to be non-Hispanic white and to have caregivers with a college or higher educational attainment than were lower-income and higher-income nonparticipants. WIC and lower-income non-WIC children were less likely to have ever been breastfed than were higher-income non-WIC children. WIC infants aged ≥6 mo were less likely to be currently breastfed than were either lower-income or higher-income nonparticipants (20). Dietary supplement use was highest in higher-income nonparticipants for infants and preschoolers (**Figure 1**).

#### Nutrient intakes of young infants (0- to 5.9-mo-olds) and older infants (6- to 11.9-mo-olds)

Regardless of WIC participation status, mean intakes of almost all micronutrients among both younger (0–5.9 mo) and older (6–11.9 mo) infants exceeded AIs, except for vitamins D and E. Only mean vitamin E intakes of younger and older infants participating in WIC were above the AIs. Younger infants participating in WIC had higher mean intakes of most micronutrients than did lower-income nonparticipants (except for vitamin A, riboflavin, vitamin C, magnesium, potassium, and sodium) or higher-income nonparticipants (except for vitamin A and sodium). Similarly, older infants participating in WIC had higher mean intakes of most micronutrients, except for riboflavin, vitamin B-6, calcium, magnesium, phosphorous, potassium, and sodium, than did lower-income nonparticipants and had higher mean intakes of almost all micronutrients, with only the exception of vitamin A, than did higher-income nonparticipants. WIC infants (<12 mo) had lower mean intakes of saturated fat as a percentage of total energy intake (percentage of energy) than did either subgroup of nonparticipants. However, total energy and sodium intakes for older infants were higher in WIC participants than in higher-income nonparticipants (Supplemental Tables 1 and 2).

In older infants, the risk of inadequate iron intakes was substantially lower in WIC participants (13%) compared with either lower-income (26%) or higher-income (34%) nonparticipants (**Table 1**). Similarly, the percentage of intakes exceeding the AI for vitamin D was higher in WIC-participating older infants (24%) than in either subgroup of nonparticipants (∼8%). The risk of inadequate zinc intake was significantly lower in WIC participants (2%) compared with higher-income nonparticipants (9%). Most older infants had usual intakes above the AIs for calcium and potassium, with a significantly larger proportion of WIC older infants meeting or exceeding the AIs compared with higher-income nonparticipants.

**TABLE 1:** Percentage of older infants aged 6–11.9 mo with intakes below or above the recommendations by WIC participation and income^1^

		Nonparticipants	
	WIC participants	Lower-income	Higher-income
Nutrients to increase	(*n *= 375)	(*n *= 169)	(*n *= 357)
Calcium,^2^ % >AI	97.7 ± 1.0	97.9 ± 2.6	93.9 ± 1.5*
Iron,^3^ % <EAR	12.6 ± 5.6	25.6 ± 6.6**	34.0 ± 2.3**
Zinc,^3^ % <EAR	1.8 ± 0.8	4.8 ± 1.5	8.9 ± 1.3**
Vitamin D,^2^ % >AI	23.7 ± 1.5	8.2 ± 5.1**	8.0 ± 2.6**
Potassium,^2^ % >AI	92.2 ± 2.0	88.3 ± 2.6	83.1 ± 1.8**

1 Values are means ± SEs. Nutrients of interest were selected on the basis of the NASEM report (8). *,**Different from WIC participants (*t* test): *P < 0.05, **P < 0.001. AI, Adequate Intake; EAR, Estimated Average Requirement; NASEM, National Academies of Sciences, Engineering, and Medicine; WIC, Special Supplemental Nutrition Program for Women, Infants, and Children.

2 Nutrient with only an AI available (21, 22).

3 Nutrient with an established EAR (23).

#### Nutrient intakes of toddlers (12- to 23.9-mo-olds)

The risk of inadequate calcium intake was higher in WIC toddlers (11%) than in lower-income nonparticipants (6%) (**Table 2**). Interestingly, the risk of inadequate iron intakes was only found among higher-income non-WIC toddlers (6%). Risks of inadequate vitamin D intake were high (>75%) across all subgroups, but WIC toddlers had a lower risk of inadequate vitamin D intake than did lower-income nonparticipants. No significant difference in DRI compliance by WIC participation group existed for zinc, fiber, or potassium. For fiber and potassium, very small proportions (<5%) of toddlers were meeting the AIs, regardless of WIC participation status. The prevalence of excessive sodium intake was notably high in both WIC participants and lower-income nonparticipants (∼45%), whereas 30% of toddlers from families with higher incomes exceeded the sodium recommendation. A higher proportion of WIC toddlers (8%) had added sugar intakes exceeding the recommendation than either category of nonparticipants (∼2%).

**TABLE 2:** Percentage of toddlers aged 12–23.9 mo with intakes below or above the recommendations by WIC participation and income^1^

		Nonparticipants	
	WIC participants	Lower-income	Higher-income
	(*n *= 380)	(*n *= 233)	(*n *= 519)
Nutrients to increase			
Calcium,^2^ % <EAR	10.7 ± 1.5	5.9 ± 1.8*	7.5 ± 2.1
Iron,^2^ % <EAR	0.0 ± 3.1	0.0 ± 6.6	6.0 ± 1.3*
Zinc,^2^ % <EAR	1.5 ± 2.6	1.1 ± 6.8	0.9 ± 1.1
Vitamin D,^2^ % <EAR	76.1 ± 1.5	82.6 ± 2.3*	78.3 ± 1.5
Fiber,^3^ % >AI	2.3 ± 2.7	2.4 ± 2.8	4.9 ± 1.3
Potassium,^3^ % >AI	2.0 ± 0.8	3.7 ± 1.7	2.5 ± 1.3
Nutrients to limit			
Sodium,^4^ % >UL	45.0 ± 0.03	46.0 ± 2.8	30.5 ± 2.0**
Added sugars,^5^ % above guideline	7.9 ± 0.7	2.4 ± 0.8**	2.0 ± 0.8**

1 Values are means ± SEs. Nutrients of interest were selected on the basis of the NASEM report (8). *,**Different from WIC participants (*t* test): *P < 0.05, **P < 0.001. AI, Adequate Intake; EAR, Estimated Average Requirement; NASEM, National Academies of Sciences, Engineering, and Medicine; UL, Tolerable Upper Intake Level; WIC, Special Supplemental Nutrition Program for Women, Infants, and Children.

2 Nutrient with an established EAR (21, 23).

3 Nutrient with only an AI available (22, 24).

4 Nutrient with an established UL (22).

5 Nutrient with an established guideline based on energy intake (<25% of energy) (24).

Mean usual intakes of nutrients were largely similar between WIC toddlers and lower-income non-WIC toddlers, whereas mean usual intakes of carbohydrate (percentage of energy), total sugar (percentage of energy), niacin, vitamin C, and sodium were higher and those of total energy, saturated fat (percentage of energy), fiber, and vitamins A and K were lower in WIC toddlers than in higher-income non-WIC toddlers (Supplemental Table 3).

#### Nutrient intakes of preschoolers (24- to 47.9-mo-olds)

No substantial difference in the percentage of children meeting the DRI recommendations by WIC participation were noted for calcium, iron, zinc, fiber, or potassium in 24- to 47.9-mo-old children (**Table 3**). The risk of inadequate vitamin D intake was significantly lower in WIC participants (80%) compared with lower-income nonparticipants (89%). WIC preschoolers had the lowest prevalence of exceeding the energy contribution from saturated fat (61%). In contrast, ∼75% and 14% of WIC preschool children consumed sodium and added sugars, respectively, at amounts above the recommendations, which is significantly higher than their higher-income counterparts.

**TABLE 3:** Percentage of preschoolers aged 24–47.9 mo with intakes below or above the recommendations by WIC participation and income^1^

		Nonparticipants	
	WIC participants	Lower-income	Higher-income
	(*n *= 161)	(*n *= 135)	(*n *= 300)
Nutrients to increase			
Calcium,^2^ % <EAR	6.6 ± 1.5	9.5 ± 3.6	8.3 ± 2.0
Iron,^2^ % <EAR	1.7 ± 3.6	3.7 ± 4.0	2.6 ± 1.1
Zinc,^2^ % <EAR	0.4 ± 4.6	0.2 ± 2.9	0.5 ± 0.8
Vitamin D,^2^ % <EAR	79.3 ± 4.1	88.7 ± 6.1*	84.2 ± 2.1
Fiber,^3^ % >AI	8.4 ± 3.5	6.6 ± 1.4	8.5 ± 2.7
Potassium,^3^ % >AI	7.0 ± 2.1	4.2 ± 2.6	3.5 ± 2.3
Nutrients to limit			
Sodium,^4^ % >UL	75.3 ± 2.6	76.3 ± 4.3	68.9 ± 2.0*
Saturated fat,^5^ % above guideline	61.0 ± 4.6	67.2 ± 4.7*	71.6 ± 2.3**
Added sugars,^6^ (% above guideline)	13.7 ± 2.2	11.1 ± 2.9	8.8 ± 2.3*

1 Values are means ± SEs. Nutrients of interest were selected on the basis of the NASEM report (8). *,**Different from WIC participants (*t* test): *P < 0.05, **P < 0.001. AI, Adequate Intake; EAR, Estimated Average Requirement; NASEM, National Academies of Sciences, Engineering, and Medicine; UL, Tolerable Upper Intake Level; WIC, Special Supplemental Nutrition Program for Women, Infants, and Children.

2 Nutrient with an established EAR (21, 23).

3 Nutrient with only an AI available (22, 24).

4 Nutrient with an established UL (22).

5 Nutrient with an established guideline based on energy intake from Dietary Guidelines for Americans (<10% of energy) (19).

6 Nutrient with an established guideline based on energy intake (<25% of energy) (24).

In WIC participants, mean usual intakes of protein (percentage of energy), vitamin C, and calcium were higher than those in lower-income nonparticipants, and mean usual intakes of thiamin, vitamin B-6, folate, vitamin B-12, vitamin C, and potassium were higher than those in higher-income nonparticipants (Supplemental Table 4). Only fat intake (percentage of energy) was lower in WIC participants than in higher-income nonparticipants.

## Discussion

The WIC program was established to help decrease poverty-related nutritional risk by providing food assistance to low-income families with young children. Despite the program's success over many decades, infants and children from low-income families still face some unique nutritional challenges that were outlined by a recent NASEM expert panel (8). The panel prioritized the nutrients that need to be increased to prevent disease and promote health, including the following: iron and zinc for older breastfed infants; iron, fiber, and potassium for toddlers; and calcium, iron, vitamin D, fiber, and potassium for 2- to 4-y-old children. The panel also prioritized the nutrients to limit, including sodium and added sugars for toddlers and sodium, added sugars, and saturated fat for 2- to 4-y-olds (8). In this study, we evaluated the risk of these nutrients relative to federal dietary recommendations, when available (21–27). This FITS 2016 report highlights similar concerns for toddlers and preschoolers as the NASEM expert committee, with additional concerns about low vitamin D intakes compared with DRIs among infants and toddlers.

The expectation of the WIC program is that it helps improve dietary intakes that may be inadequate due to lack of money by providing a food package and nutrition education. The current WIC package provides iron-fortified infant formula to partially or not-breastfeeding younger infants, iron-fortified infant formula and infant foods (cereal, fruit, and vegetables) for partially or not-breastfeeding older infants, and infant foods and infant meat for fully breastfeeding older infants. The WIC food package for 1- to 4-y-olds includes vitamin D–fortified milk, iron-fortified breakfast cereal, eggs, whole-grain breads, legumes or peanut butter, fruit and vegetables, and vitamin C–rich juice (7). Thus, we assumed that the nutrient intakes of WIC participants would be more like those of higher-income children and that those at the greatest nutritional risk would be lower-income nonparticipants, with all other factors being equal. However, all other factors were not equal in the sample. In particular, the use of dietary supplements was substantially higher in higher-income nonparticipants, so it was not possible to test such an assumption. Therefore, we compared only the nutrient intakes from the diets of WIC participants with those of either lower-income or higher-income nonparticipants, because the WIC package provides foods and beverages but not dietary supplements.

In infants (aged 0–11.9 mo), WIC participation was associated with a higher mean nutrient intake of most micronutrients, with lower saturated fat as a percentage of energy when compared with those not participating in WIC, regardless of eligibility. The WIC program appears to be especially beneficial to older infants (aged 6–11.9 mo), particularly in that it is associated with a lower risk of iron and zinc inadequacy. This finding on iron is consistent not only with the WIC program goals but also with previous reports (28–30), which may be attributed to a higher likelihood of consumption of iron-fortified cereal, infant formula, and baby-food meats by WIC participants in FITS 2016 (20). However, 13% of WIC-participating older infants were still at risk of inadequate iron intakes, compared with 1% among WIC infants aged 7–11.9 mo in FITS 2002 (16). This may be partly attributable to increased breastfeeding rate (31), decreased infant cereal consumption (31), and low infant meat consumption (20) in this age group. Iron stores at birth may meet iron needs during the first 6 mo, but iron supplementation through complementary feeding is suggested from ages 4 to 6 mo (32, 33). The results highlight the continued need for education on the importance of complementary foods containing iron. Furthermore, in older infants, WIC participants had better nutritional risk profiles in general when compared with recommendations for all nutrients examined, even though only iron and zinc were listed by NASEM as priority nutrients in this age group.

In toddlers (aged 12–23.9 mo), the priority nutrients are iron, potassium, and fiber as nutrients to increase and sodium and added sugars as nutrients to limit. Paradoxically, in this age group, iron intakes were only of concern for the higher-income nonparticipants. The results might be different if iron intake from dietary supplements was included, but iron supplement use was very low (5%) among this age group (34). Meanwhile, WIC participation was associated with higher compliance with sodium and added-sugars guidelines, with no significant differences noted in compliance with potassium or fiber guidelines. A FITS 2016 food-based analysis (20) suggested that WIC toddlers tended to have a higher consumption of fruit-flavored drinks and sweetened beverages that may be contributing added sugars, but it is not clear which food groups are responsible for differences in sodium intake.

For preschoolers, the same nutrients are prioritized as for toddlers, plus calcium, vitamin D, and saturated fat. The revised food packages limit milk fat content to ≤1% for children aged >2 y. As a consequence, several regional studies have reported reduced saturated fat intakes, with a concomitant shift toward lower-fat milks (2%, 1%, or nonfat), in WIC children aged ≥2 y after the food package change (35–37). In FITS 2016, 1% low-fat milk was the most commonly consumed milk among WIC children aged 24–47.9 mo, whereas 2% reduced-fat milk was more prevalently consumed among both subgroups of nonparticipants (20), which may contribute to a lower percentage of WIC children aged 24–47.9 mo exceeding the recommendations for saturated fat intakes. In terms of compliance with recommendations for sodium and added sugars (percentage of energy), WIC preschoolers were similar to lower-income nonparticipants but were less likely to comply with the recommendations than their higher-income counterparts. Added sugar is an area of controversy, especially in young children (38). We used the NASEM guidelines of 25% of energy from added sugar as the benchmark, but other authoritative bodies have endorsed <10% of total energy intake from added sugars or free sugars (19, 39). Certainly, a much higher prevalence of exceeding added sugar would be reported on the basis of the FITS 2016 data if 10% were used as the guideline. However, the 10% recommendation may be very difficult to achieve and experts differ on the strength of evidence supporting such a recommendation (40–42). NHANES 2011–2012 data suggested that 80% of WIC participants (ages 2–4 y) exceeded the 10% recommendation (8).

Although vitamin D is only highlighted as a nutrient of concern in those aged ≥24 mo, most infants and young children failed to meet vitamin D recommendations, regardless of WIC participation status. This study as well as national and program-specific reports have shown that most US infants and children have suboptimal vitamin D intakes from foods and beverages (43, 44). The American Academy of Pediatrics recommends routine vitamin D supplementation for breastfed infants (45); with more than three-quarters of toddlers and preschoolers at risk of vitamin D inadequacy from foods alone, supplementation may be warranted in older children as well. In this supplement issue, Bailey et al. (34) showed that, for users of vitamin D supplements, the supplements added an average of ∼10 μg vitamin D/d, which corresponds to the amount recommended as the AI (<12 mo) or EAR (≥12 mo), but when vitamin D supplement users and nonusers were combined, most infants and young children still failed to meet recommendations. Nonetheless, it is important to note that WIC participation was consistently associated with better vitamin D intakes compared with lower-income nonparticipants across all age groups.

Some limitations should be noted in the interpretation of the present study. Dietary information was reported by parents or caregivers and may not fully represent the actual intake of the child. However, 24-h recalls were conducted by trained interviewers with the use of the automated multiple-pass approach to minimize this inaccuracy. Usual nutrient intakes were estimated with the use of the NCI method, which relies on the assumption that a 24-h dietary recall is unbiased for single-day intake; this may or may not be the case (46, 47). Biomarkers of nutritional status would be preferable, if they exist, and would be especially helpful in evaluating the iron and vitamin D findings of this report. We compared WIC participants with lower-income (likely income-eligible) nonparticipants, but the factors related to the decision to participate in WIC (48, 49) were not assessed. In addition, approximately half of WIC participants were also participating in SNAP. SNAP provides benefits to buy food to low-income individuals and families, and it is possible to participate in both WIC and SNAP. Thus, it is more difficult to attribute associations here exclusively to WIC, because infant formula and most foods can also be purchased with the use of SNAP benefits. However, WIC participation has been found to increase nutrient intakes of young children, whereas SNAP had no additional benefit in those already participating in WIC (28). In the 24-h recall, we did not identify which foods were provided by WIC. Finally, we evaluated differences between WIC participants and others on the basis of nutrient intakes from foods and beverages alone because dietary supplements are not part of the WIC package. This may underestimate the nutrient intakes, especially for children from higher-income families because they consumed more dietary supplements than both lower-income groups. Future work could investigate total nutrient intakes from both diet and supplements by WIC participation status and identify major sources of priority nutrients to target for improvement, such as breast milk, infant formula, and supplements.

Although we acknowledge that there is great interest in comparing the impact of WIC food package revision on usual nutrient intakes, we could not immediately compare the results from FITS 2016 with FITS 2008 or FITS 2002 because the sampling frames were different, WIC participants and nonparticipants were classified in a different way, and usual nutrient intakes were estimated by using a different methodology (PC-SIDE from Iowa State University). In fact, the FITS 2008 study only reported on food-group consumption related to WIC participation status rather than nutrient intakes (9). Even looking outside FITS, there are few data on the impact of the WIC food package change on nutrient intakes (28, 50). Instead, the impact has been measured by improvements in the Healthy Eating Index score (51) or food-group consumption (35, 36, 52). This is a priority area that would benefit from future exploration. Nonetheless, the findings may be useful for enhancing understanding of the dietary intakes of WIC participants, identifying the remaining dietary issues after the 2009 WIC food package revision, and comparing findings against the latest NASEM committee recommendations for further changes in the WIC food package. The present study provides the most comprehensive and recent estimates on nutrient intakes of WIC infants and young children, updating and extending the previous national-scope studies that were conducted before the 2009 WIC food package revision (16, 28, 44, 53, 54). To our knowledge, this is the first study to report usual dietary intakes of infants aged 0–5.9 mo by WIC participation status.

In conclusion, WIC participation may be nutritionally beneficial (e.g., participants had dietary intakes closer to recommendations), especially for iron (ages 6–23.9 mo), zinc (ages 6–11.9 mo), saturated fat (ages 24–47.9 mo), and vitamin D (all age groups). Nevertheless, it is notable that 13% of WIC infants (6–11.9 mo) were still not meeting iron recommendations. No substantial differences in nutritional risk profiles were noted for calcium, iron, zinc, potassium, or fiber between WIC and non-WIC toddlers and preschoolers. However, of concern, WIC participation among toddlers and preschoolers was associated with an increased prevalence of exceeding the energy recommendations for added sugar and sodium intakes. Future work is needed to determine how to best support food and beverage choices to reduce added sugar and sodium consumption in all children, but especially among those in lower-income families. In addition, greater effort to encourage more consumption of fiber, vitamin D, iron, and potassium and less consumption of saturated fat among WIC children as well as non-WIC children is warranted.

## Supplementary Material

Supplement Tables

## Notes

Published in a supplement to *The Journal of Nutrition*. Select contents of this supplement were presented at the Experimental Biology 2017 conference in a session titled, “Informing B-24 Dietary Guidelines: Findings from the New Feeding Infants and Toddlers Study 2016,” held in Chicago, IL, April 22, 2017. Supplement Coordinators were William Dietz, Milken School of Public Health at the George Washington University and Andrea S. Anater, RTI International. The FITS research was funded by the Nestle Research Centre (Lausanne, Switzerland) through a contract with RTI International and its subcontractor, the University of Minnesota. Dr. Dietz is a compensated consultant to RTI on this project. The article contents are the responsibility of the authors and do not necessarily represent the opinions or recommendations of Nestle. Publication costs for this supplement were defrayed in part by the payment of page charges. This publication must therefore be hereby marked “advertisement” in accordance with 18 USC section 1734 solely to indicate this fact. The opinions expressed in this publication are those of the authors and are not attributable to the sponsors or the publisher, Editor, or Editorial Board of *The Journal of Nutrition*. Supported by Nestlé Research Center, Vers-Chez-les-Blanc, Route du Jorat 57, Case Postale 44, 1000 Lausanne-26, Switzerland.

Author disclosures: SJ, DJC, and HAE-M, Dr. Dwyer is a member of the Scientific Advisory Boards of Conagra Foods and McCormick Spice, has served as a consultant for Gerber/Nestle, and has received partial travel expenses to moderate a symposium at the International Congress of Nutrition in Buenos Aires Argentina in 2017 from the International Alliance of Dietary Supplement Associations. She holds stock in several food and drug companies. Bailey is a consultant to RTI International. ALE is an employee of the Nestlé Research Center (funding source). JTD is a member of the Scientific Advisory Boards of Conagra Foods and McCormick Spice, has served as a consultant for Gerber/Nestle, and has received partial travel expenses to moderate a symposium at the International Congress of Nutrition in Buenos Aires Argentina in 2017 from the International Alliance of Dietary Supplement Associations. She holds stock in several food and drug companies. RLB was a scientific consultant of RTI International for the FITS 2016 project.

Supplemental Tables 1–4 are available from the “Supplementary data” link in the online posting of the article and from the same link in the online table of contents at https://academic.oup.com/jn/.

Abbreviations used:

AI: Adequate Intake
EAR: Estimated Average Requirement
FITS: Feeding Infants and Toddlers Study
NASEM: National Academies of Sciences, Engineering, and Medicine
NCI: National Cancer Institute
SNAP: Supplementary Nutrition Assistance Program
WIC: Special Supplemental Nutrition Program for Women, Infants and Children
