# Supplementary material for: Usual Nutrient Intakes from the Diets of US Children by WIC Participation and Income: Findings from the Feeding Infants and Toddlers Study (FITS) 2016
Source: J Nutr. 2018 Jun 5;148(Suppl 3):1567S–1574S. doi: 10.1093/jn/nxy059 (PMC6126631; doi:10.1093/jn/nxy059)
Supplement: Supplement Tables [file nxy059_supplement_tables.docx]

**Supplemental Table 1. Usual dietary intake from food and beverages by WIC participation status for
young infants 0-5.9 mo, Feeding Infants and Toddlers Study 2016 (*n*=600)^1^**

| **Macronutrients^2^** | **DRI Values** | | **WIC participants (*n*=245)** | **Nonparticipants** | |
| --- | --- | --- | --- | --- | --- |
|  |  |  |  | **Lower-income**  **(*n*=104)** | **Higher-income**  **(*n*=251)** |
|  | **AI** | **UL** |  |  |  |
| Energy (kcal/d) | – | – | 671 ± 10.9 | 662 ± 19.5 | 637 ± 10.0 |
| Fat (g/d) | 31 | – | 37 ± 0.7 | 37 ± 1.3 | 37 ± 0.7 |
| Saturated fat (g/d) | – | – | 15 ± 0.4 | 16 ± 0.5 | 16 ± 0.4 |
| Carbohydrate (g/d) | 60 | – | 75 ± 1.4 | 75 ± 1.4 | 69 ± 1.2^*^ |
| Protein (g/d) | 9.1 | – | 14 ± 0.3 | 13 ± 0.5 | 12 ± 0.2^*^ |
| Dietary fiber (g/d) | – | – | 1.5 ± 0.1 | 1.4 ± 0.1 | 0.8 ± 0.1^*^ |
| Fat (% kcal) | – | – | 48 ± 0.3 | 49 ± 0.3 | 51 ± 0.3^*^ |
| Saturated fat (% kcal) | – | – | 20 ± 0.2 | 21 ± 0.2^*^ | 22 ± 0.2^*^ |
| Carbohydrate (% kcal) | – | – | 43 ± 0.3 | 43 ± 0.4 | 41 ± 0.4^*^ |
| Total Sugar (% kcal) | – | – | 34 ± 0.4 | 38 ± 0.5^*^ | 37 ± 0.3^*^ |
| Protein (% kcal) | – | – | 8.1 ± 0.1 | 7.7 ± 0.1 | 7.4 ± 0.1^*^ |
| **Micronutrients** | | | | | |
| Vitamin A (μg RAE/d)^3^ | 400 | 600 | 610 ± 14.2 | 622 ± 13.8 | 582 ± 10.0 |
| Thiamin (mg/d)^4^ | 0.2 | – | 0.4 ± 0.01 | 0.3 ± 0.01^*^ | 0.3 ± 0.01^*^ |
| Riboflavin (mg/d)^4^ | 0.3 | – | 0.7 ± 0.02 | 0.6 ± 0.03 | 0.5 ± 0.02^*^ |
| Niacin (mg/d)^4^ | 2 | – | 5.5 ± 0.2 | 4.1 ± 0.2^*^ | 3.4 ± 0.1^*^ |
| Vitamin B-6 (mg/d)^4^ | 0.1 | – | 0.3 ± 0.01 | 0.3 ± 0.01^*^ | 0.2 ± 0.01^*^ |
| Folate (μg DFE/d)^4,5^ | 65 | – | 130 ± 3.8 | 101 ± 4.0^*^ | 86 ± 2.1^*^ |
| Vitamin B-12 (μg/d)^4^ | 0.4 | – | 1.6 ± 0.1 | 1.3 ± 0.1^*^ | 1.1 ± 0.05^*^ |
| Vitamin C (mg/d)^6^ | 40 | – | 77 ± 1.8 | 77 ± 2.1 | 63 ± 1.6^*^ |
| Vitamin D (μg/d)^7^ | 10 | 25 | 6.5 ± 0.2 | 4.5 ± 0.3^*^ | 3.3 ± 0.2^*^ |
| Vitamin E (mg/d)^6^ | 4 | – | 4.3 ± 0.1 | 3.0 ± 0.2^*^ | 2.1 ± 0.1^*^ |
| Vitamin K (μg/d)^3^ | 2 | – | 32 ± 1.2 | 21 ± 1.4^*^ | 13 ± 0.6^*^ |
| Calcium (mg/d)^7^ | 200 | 1,000 | 517 ± 14.1 | 447 ± 17.1^*^ | 398 ± 11.3^*^ |
| Iron (mg/d)^3^ | 0.27 | 40 | 7.8 ± 0.3 | 5.1 ± 0.3^*^ | 3.1 ± 0.1^*^ |
| Magnesium (mg/d)^8^ | 30 | – | 53 ± 1.4 | 48 ± 2.0 | 40 ± 1.1^*^ |
| Phosphorus (mg/d) ^8^ | 100 | – | 270 ± 7.6 | 229 ± 10.2^*^ | 200 ± 6.1^*^ |
| Potassium (mg/d)^9^ | 400 | – | 689 ± 14.7 | 661 ± 26.8 | 586 ± 13.2^*^ |
| Sodium (mg/d)^9^ | 120 | – | 222 ± 6.2 | 212 ± 11.1 | 196 ± 5.9 |
| Zinc (mg/d)^3^ | 2 | 4 | 4.4 ± 0.1 | 3.7 ± 0.2^*^ | 2.8 ± 0.1^*^ |

AI, Adequate Intake; DFE, dietary folate equivalents; DRI, Dietary Reference Intake; RAE, retinol activity equivalents; WIC, Special Supplemental Nutrition Program for Women, Infants, and Children.

^1^ Values are mean ± SEs. ^*^ Significantly different from WIC participants by multiple t tests at Bonferroni-corrected *P* value of 0.002.

^2^ All macronutrient DRIs are from 2005 DRI book for energy, carbohydrates, etc. (24).

^3^ DRIs are from 2001 DRI book for vitamin A, vitamin K, and various metals including iron and zinc (23).

^4^ DRIs are from 1998 DRI book for thiamin, riboflavin, niacin, vitamin B6, folate, vitamin B12, and others(25).

^5^ Synthetic Folate (folic acid) from supplements multiplied by 1.6 to convert it to the dietary folate equivalent metric.

^6^ DRIs are from 2000 DRI book for vitamin C, vitamin E, selenium, and carotenoids (26).

^7^ DRIs are from 2011 DRI book for calcium and vitamin D (21).

^8^ DRIs are from 1997 DRI book for calcium, phosphorus, magnesium, vitamin D, and fluoride (27).

^9^ DRIs are from 2005 DRI book for water, potassium, sodium, chloride, and sulfate (22).

**Supplemental Table 2. Usual dietary intake from food and beverages by WIC participation status for**

**older infants 6-11.9 mo, Feeding Infants and Toddlers Study 2016 (*n*=901)^1^**

| **Macronutrients^2^** | **DRI Values** | | | | **WIC participants (*n*=375)** | **Nonparticipants** | |
| --- | --- | --- | --- | --- | --- | --- | --- |
|  | **AMDR** | | **AI** | **UL** |  | **Lower-income (*n*=169)** | **Higher-income**  **(*n*=357)** |
| Energy (kcal/d) | – | | – | – | 873 ± 11.4 | 861 ± 19.8 | 796 ± 10.6^*^ |
| Fat (g/d) | – | | 30 | – | 38 ± 0.6 | 39 ± 1.1 | 38 ± 0.6 |
| Saturated fat (g/d) | – | | – | – | 15 ± 0.3 | 16 ± 0.4 | 16 ± 0.3 |
| Carbohydrate (g/d) | – | | 95 | – | 116 ± 1.6 | 110 ± 2.5 | 100 ± 1.5^*^ |
| Protein (g/d) | – | | – | – | 21.0 ± 0.4 | 21 ± 0.6 | 19 ± 0.3^*^ |
| Dietary fiber (g/d) | – | | – | – | 6.6 ± 0.2 | 6.4 ± 0.2 | 5.8 ± 0.2^*^ |
| Fat (% kcal) | – | | – | – | 39 ± 0.2 | 40 ± 0.3 | 41 ± 0.3^*^ |
| Saturated fat (% kcal) | – | | – | – | 15 ± 0.2 | 16 ± 0.2^*^ | 17 ± 0.2^*^ |
| Carbohydrate (% kcal) | – | | – | – | 52 ± 0.3 | 50 ± 0.3^*^ | 48 ± 0.3^*^ |
| Total Sugar (% kcal) | – | | – | – | 33 ± 0.3 | 33 ± 0.4 | 34 ± 0.3 |
| Protein (% kcal) | – | | – | – | 9.4 ± 0.1 | 9.8 ± 0.1 | 9.4 ± 0.1 |
| **Micronutrients** | | **EAR** | **AI** | **UL** |  |  |  |
| Vitamin A (μg RAE/d)^3^ | – | | 500 | 600 | 806 ± 13.7 | 739 ± 12.6^*^ | 789 ± 10.2 |
| Thiamin (mg/d)^4^ | – | | 0.3 | – | 0.7 ± 0.02 | 0.6 ± 0.02^*^ | 0.6 ± 0.01^*^ |
| Riboflavin (mg/d)^4^ | – | | 0.4 | – | 1.1 ± 0.02 | 1.0 ± 0.03 | 0.9 ± 0.02^*^ |
| Niacin (mg/d)^4^ | – | | 4 | – | 9.2 ± 0.2 | 7.8 ± 0.2^*^ | 7.2 ± 0.1^*^ |
| Vitamin B-6 (mg/d)^4^ | – | | 0.3 | – | 0.7 ± 0.01 | 0.7 ± 0.02 | 0.6 ± 0.01^*^ |
| Folate (μg DFE/d)^4,5^ | – | | 80 | – | 221 ± 4.9 | 190 ± 5.6^*^ | 168 ± 3.2^*^ |
| Vitamin B-12 (μg/d)^4^ | – | | 0.5 | – | 2.3 ± 0.1 | 2.0 ± 0.1^*^ | 1.8 ± 0.1^*^ |
| Vitamin C (mg/d)^6^ | – | | 50 | – | 101 ± 1.8 | 85 ± 1.8^*^ | 80 ± 1.6^*^ |
| Vitamin D (μg/d)^7^ | – | | 10 | 38 | 7.4 ± 0.2 | 5.6 ± 0.2^*^ | 4.8 ± 0.2^*^ |
| Vitamin E (mg/d)^6^ | – | | 5 | – | 6.3 ± 0.2 | 4.8 ± 0.2^*^ | 4.4 ± 0.1^*^ |
| Vitamin K (μg/d)^3^ | – | | 2.5 | – | 55 ± 1.5 | 41 ± 2.1^*^ | 39 ± 1.4^*^ |
| Calcium (mg/d)^7^ | – | | 260 | 1,500 | 672 ± 14.1 | 612 ± 17.0 | 561 ± 12.5^*^ |
| Iron (mg/d)^3^ | 6.9 | | – | 40 | 14 ± 0.3 | 10 ± 0.3^*^ | 9.4 ± 0.3^*^ |
| Magnesium (mg/d)^8^ | – | | 75 | – | 103 ± 1.9 | 101 ± 3.1 | 88 ± 1.8^*^ |
| Phosphorus (mg/d) ^8^ | – | | 275 | – | 425 ± 8.9 | 427 ± 13.0 | 368 ± 8.2^*^ |
| Potassium (mg/d)^9^ | – | | 700 | – | 1158 ± 18.9 | 1161 ± 32.6 | 1019 ± 18.2^*^ |
| Sodium (mg/d)^9^ | – | | 370 | – | 416 ± 9.3 | 436 ± 16.4 | 349 ± 8.3^*^ |
| Zinc (mg/d)^3^ | 2.5 | | – | 5 | 6.3 ± 0.1 | 5.3 ± 0.2^*^ | 4.7 ± 0.1^*^ |

AI, Adequate Intake; DFE, dietary folate equivalents; DRI, Dietary Reference Intake; EAR, Estimated Average Requirement; RAE, retinol activity equivalents; WIC, Special Supplemental Nutrition Program for Women, Infants, and Children.

^1^ Values are mean ± SEs. ^*^ Significantly different from WIC participants by multiple t tests at Bonferroni-corrected *P* value of 0.002.

^2^ All macronutrient DRIs are from 2005 DRI book for energy, carbohydrates, etc. (24).

^3^ DRIs are from 2001 DRI book for vitamin A, vitamin K, and various metals including iron and zinc (23).

^4^ DRIs are from 1998 DRI book for thiamin, riboflavin, niacin, vitamin B6, folate, vitamin B12, and others (25).

^5^ Synthetic Folate (folic acid) from supplements multiplied by 1.6 to convert it to the dietary folate equivalent metric.

^6^ DRIs are from 2000 DRI book for vitamin C, vitamin E, selenium, and carotenoids (26).

^7^ DRIs are from 2011 DRI book for calcium and vitamin D (21).

^8^ DRIs are from 1997 DRI book for calcium, phosphorus, magnesium, vitamin D, and fluoride (27).

^9^ DRIs are from 2005 DRI book for water, potassium, sodium, chloride, and sulfate (22).

**Supplemental Table 3. Usual dietary intake from food and beverages by WIC participation status for
toddlers 12-23.9 mo, Feeding Infants and Toddlers Study 2016 (*n*=1,132)^1^**

| **Macronutrients^2^** | **DRI Values** | | | **WIC participants (*n*=380)** | **Nonparticipants** | |
| --- | --- | --- | --- | --- | --- | --- |
|  | **AMDR** | **AI** | **UL** |  | **Lower-income (*n*=233)** | **Higher-income**  **(*n*=519)** |
| Energy (kcal/d) | – | – | – | 1188 ± 15.3 | 1237 ± 23.8 | 1125 ± 12.2^*^ |
| Fat (g/d) | – | – | – | 44 ± 0.7 | 48 ± 1.0 | 43 ± 0.6 |
| Saturated fat (g/d) | – | – | – | 17 ± 0.3 | 19 ± 0.4 | 17 ± 0.3 |
| Carbohydrate (g/d) | – | – | – | 156 ± 2.2 | 159 ± 3.0 | 146 ± 1.6^*^ |
| Protein (g/d) | – | – | – | 46 ± 0.8 | 47 ± 1.0 | 45 ± 0.6 |
| Dietary fiber (g/d) | – | 19 | – | 9.5 ± 0.2 | 9.9 ± 0.3 | 10.8 ± 0.2^*^ |
| Fat (% kcal) | 30–40 | – | – | 32 ± 0.3 | 34 ± 0.2^*^ | 33 ± 0.2 |
| Saturated fat (% kcal) | – | – | – | 13 ± 0.2 | 13 ± 0.1 | 14 ± 0.1^*^ |
| Carbohydrate (% kcal) | 45–65 | – | – | 52 ± 0.3 | 51 ± 0.3 | 50 ± 0.2^*^ |
| Total Sugar (% kcal) | – | – | – | 29 ± 0.3 | 28 ± 0.4 | 27 ± 0.2^*^ |
| Protein (% kcal) | 5–20 | – | – | 16 ± 0.2 | 16 ± 0.2 | 16 ± 0.1 |
| **Micronutrients** | **EAR** | **AI** | **UL** |  |  |  |
| Vitamin A (μg RAE/d)^3^ | 210 | – | 600 | 560 ± 10.9 | 576 ± 8.7 | 635 ± 7.3^*^ |
| Thiamin (mg/d)^4^ | 0.4 | – | – | 1.0 ± 0.02 | 1.1 ± 0.02 | 1.0 ± 0.01 |
| Riboflavin (mg/d)^4^ | 0.4 | – | – | 1.6 ± 0.03 | 1.6 ± 0.04 | 1.6 ± 0.03 |
| Niacin (mg/d)^4^ | 5 | – | – | 12 ± 0.2 | 12 ± 0.3 | 11 ± 0.2^*^ |
| Vitamin B-6 (mg/d)^4^ | 0.4 | – | – | 1.1 ± 0.02 | 1.1 ± 0.02 | 1.1 ± 0.02 |
| Folate (μg DFE/d)^4,5^ | 120 | – | 300 | 302 ± 6.3 | 298 ± 7.2 | 289 ± 4.3 |
| Vitamin B-12 (μg/d)^4^ | 0.7 | – | – | 3.6 ± 0.1 | 3.5 ± 0.1 | 3.7 ± 0.1 |
| Vitamin C (mg/d)^6^ | 13 | – | 400 | 67 ± 1.3 | 64 ± 1.2 | 59 ± 1.1^*^ |
| Vitamin D (μg/d)^7^ | 10 | – | 63 | 7.4 ± 0.2 | 6.9 ± 0.2 | 7.1 ± 0.2 |
| Vitamin E (mg/d)^6^ | 5 | – | 200 | 4.7 ± 0.1 | 4.9 ± 0.2 | 4.8 ± 0.1 |
| Vitamin K (μg/d)^3^ | – | 30 | – | 37 ± 1.1 | 44 ± 1.9^*^ | 44 ± 1.3^*^ |
| Calcium (mg/d)^7^ | 500 | – | 2,500 | 872 ± 17.2 | 902 ± 19.3 | 927 ± 15.0 |
| Iron (mg/d)^3^ | 3 | – | 40 | 9.1 ± 0.3 | 8.7 ± 0.3 | 8.7 ± 0.2 |
| Magnesium (mg/d)^8^ | 65 | – | 65 | 169 ± 2.8 | 173 ± 4.1 | 174 ± 2.7 |
| Phosphorus (mg/d) ^8^ | 380 | – | 3,000 | 865 ± 15.4 | 887 ± 19.3 | 886 ± 13.1 |
| Potassium (mg/d)^9^ | – | 3,000 | – | 1732 ± 27.0 | 1793 ± 38.7 | 1734 ± 23.6 |
| Sodium (mg/d)^9^ | – | 1,000 | 1,500 | 1549 ± 33.6 | 1549 ± 41.9 | 1318 ± 21.7^*^ |
| Zinc (mg/d)^3^ | 2.5 | – | 7 | 6.5 ± 0.1 | 6.5 ± 0.1 | 6.7 ± 0.1 |

AI, Adequate Intake; AMDR, Acceptable Macronutrient Distribution Range; DFE, dietary folate equivalents; DRI, Dietary Reference Intake; EAR, Estimated Average Requirement; RAE, retinol activity equivalents; WIC, Special Supplemental Nutrition Program for Women, Infants, and Children.

^1^ Values are mean ± SEs. ^*^ Significantly different from WIC participants by multiple t tests at Bonferroni-corrected *P* value of 0.002.

^2^ All macronutrient DRIs are from 2005 DRI book for energy, carbohydrates, etc. (24).

^3^ DRIs are from 2001 DRI book for vitamin A, vitamin K, and various metals including iron and zinc (23).

^4^ DRIs are from 1998 DRI book for thiamin, riboflavin, niacin, vitamin B6, folate, vitamin B12, and others (25).

^5^ Synthetic Folate (folic acid) from supplements multiplied by 1.6 to convert it to the dietary folate equivalent metric.

^6^ DRIs are from 2000 DRI book for vitamin C, vitamin E, selenium, and carotenoids (26).

^7^ DRIs are from 2011 DRI book for calcium and vitamin D (21).

^8^ DRIs are from 1997 DRI book for calcium, phosphorus, magnesium, vitamin D, and fluoride (27).

^9^ DRIs are from 2005 DRI book for water, potassium, sodium, chloride, and sulfate (22).

**Supplemental Table 4. Usual dietary intake from food and beverages by WIC participation status for
preschoolers 24-47.9 mo, Feeding Infants and Toddlers Study 2016 (*n*=596)^1^**

| **Macronutrients^2^** | **DRI** | | | | **WIC Participants**  **(*n*=161)** | **Nonparticipants** | |
| --- | --- | --- | --- | --- | --- | --- | --- |
|  | **AMDR** | | **AI** | **UL** |  | **Lower-income**  **(*n*=135)** | **Higher-income**  **(*n*=300)** |
| Energy (kcal/d) | – | | – | – | 1442 ± 28.5 | 1444 ± 36.0 | 1339 ± 19.5 |
| Fat (g/d) | – | | – | – | 50 ± 1.1 | 52 ± 1.5 | 49 ± 0.9 |
| Saturated fat (g/d) | – | | – | – | 18 ± 0.5 | 18 ± 0.5 | 18 ± 0.3 |
| Carbohydrate (g/d) | – | | – | – | 198 ± 4.2 | 198 ± 4.8 | 180 ± 2.6^*^ |
| Protein (g/d) | – | | – | – | 56 ± 1.4 | 52 ± 1.5 | 52 ± 0.9 |
| Dietary fiber (g/d) | – | | 19 | – | 12 ± 0.4 | 12 ± 0.4 | 12 ± 0.3 |
| Fat (% kcal) | 30-40 | | – | – | 30 ± 0.4 | 31 ± 0.3 | 32 ± 0.3**^*^** |
| Saturated fat (% kcal) | – | | – | – | 11 ± 0.2 | 11 ± 0.1 | 12 ± 0.2 |
| Carbohydrate (% kcal) | 45-65 | | – | – | 54 ± 0.4 | 54 ± 0.4 | 52 ± 0.3 |
| Total Sugar (% kcal) | – | | – | – | 29 ± 0.5 | 28 ± 0.5 | 27 ± 0.3 |
| Protein (% kcal) | 5-20 | | – | – | 16 ± 0.2 | 15 ± 0.2^*^ | 16 ± 0.1 |
| **Micronutrients** | | **EAR** | **AI** | **UL** |  |  |  |
| Vitamin A (μg RAE/d)^3^ | 210 | | – | 600 | 588 ± 17.5 | 565 ± 11.8 | 597 ± 9.4 |
| Thiamin (mg/d)^4^ | 0.4 | | – | – | 1.3 ± 0.03 | 1.3 ± 0.03 | 1.2 ± 0.02**^*^** |
| Riboflavin (mg/d)^4^ | 0.4 | | – | – | 1.8 ± 0.05 | 1.7 ± 0.05 | 1.6 ± 0.04 |
| Niacin (mg/d)^4^ | 5.0 | | – | – | 15 ± 0.4 | 15 ± 0.5 | 14 ± 0.3 |
| Vitamin B-6 (mg/d)^4^ | 0.4 | | – | – | 1.4 ± 0.04 | 1.3 ± 0.04 | 1.2 ± 0.02**^*^** |
| Folate (μg DFE/d)^4,5^ | 120 | | – | 300 | 388 ± 12.2 | 384 ± 11.8 | 342 ± 6.7**^*^** |
| Vitamin B-12 (μg/d)^4^ | 0.7 | | – | – | 4.2 ± 0.1 | 3.7 ± 0.1 | 3.6 ± 0.1**^*^** |
| Vitamin C (mg/d)^6^ | 13 | | – | 400 | 81 ± 2.4 | 67 ± 1.8^*^ | 63 ± 1.5**^*^** |
| Vitamin D (μg/d)^7^ | 10 | | – | 63 | 7.0 ± 0.3 | 6.0 ± 0.3 | 6.2 ± 0.2 |
| Vitamin E (mg/d)^6^ | 5.0 | | – | 200 | 5.6 ± 0.2 | 5.8 ± 0.3 | 5.9 ± 0.2 |
| Vitamin K (μg/d)^3^ | – | | 30 | – | 48 ± 2.1 | 53 ± 3.0 | 49 ± 1.9 |
| Calcium (mg/d)^7^ | 500 | | – | 2,500 | 966 ± 28.8 | 840 ± 24.4^*^ | 916 ± 19.8 |
| Iron (mg/d)^3^ | 3.0 | | – | 40 | 11 ± 0.5 | 10 ± 0.4 | 9.5 ± 0.3 |
| Magnesium (mg/d)^8^ | 65 | | – | 65 | 202 ± 5.0 | 187 ± 5.8 | 194 ± 3.9 |
| Phosphorus (mg/d) ^8^ | 380 | | – | 3,000 | 1020 ± 27.2 | 933 ± 26.4 | 979 ± 18.9 |
| Potassium (mg/d)^9^ | – | | 3,000 | – | 2017 ± 47.8 | 1822 ± 51.8 | 1820 ± 33.0**^*^** |
| Sodium (mg/d)^9^ | – | | 1,000 | 1,500 | 2155 ± 72.1 | 2128 ± 71.9 | 1900 ± 39.9 |
| Zinc (mg/d)^3^ | 2.5 | | – | 7 | 7.8 ± 0.2 | 7.5 ± 0.2 | 7.3 ± 0.2 |

AI, Adequate Intake; AMDR, Acceptable Macronutrient Distribution Range; DFE, dietary folate equivalents; DRI, Dietary Reference Intake; EAR, Estimated Average Requirement; RAE, retinol activity equivalents; WIC, Special Supplemental Nutrition Program for Women, Infants, and Children.

^1^ Values are mean ± SEs. ^*^ Significantly different from WIC participants by multiple t tests at Bonferroni-corrected *P* value of 0.002.

^2^ All macronutrient DRIs are from 2005 DRI book for energy, carbohydrates, etc. (24).

^3^ DRIs are from 2001 DRI book for vitamin A, vitamin K, and various metals including iron and zinc (23).

^4^ DRIs are from 1998 DRI book for thiamin, riboflavin, niacin, vitamin B6, folate, vitamin B12, and others (25).

^5^ Synthetic Folate (folic acid) from supplements multiplied by 1.6 to convert it to the dietary folate equivalent metric.

^6^ DRIs are from 2000 DRI book for vitamin C, vitamin E, selenium, and carotenoids (26).

^7^ DRIs are from 2011 DRI book for calcium and vitamin D (21).

^8^ DRIs are from 1997 DRI book for calcium, phosphorus, magnesium, vitamin D, and fluoride (27).

^9^ DRIs are from 2005 DRI book for water, potassium, sodium, chloride, and sulfate (22).
